# Second Primary Cancer Among Patients With Papillary Thyroid Carcinoma Following the Chernobyl Disaster

**DOI:** 10.1001/jamanetworkopen.2023.29559

**Published:** 2023-08-17

**Authors:** Anas Taha, Stephanie Taha-Mehlitz, Eldar A. Nadyrov, Dmitry Zinovkin, Ilya Veyalkin, Leonid Levin, Md Zahidul I. Pranjol, Nathaniel Melling, Michael D. Honaker, Philippe C. Cattin, Ralph A. Schmid

**Affiliations:** 1 Department of Biomedical Engineering, Faculty of Medicine, University of Basel, Allschwil, Switzerland; 2 Clarunis, Department of Visceral Surgery, University Center for Gastrointestinal and Liver Diseases, St Clara Hospital and University Hospital Basel, Basel, Switzerland; 3 Department of Pathology, Gomel State Medical University, Gomel, Belarus; 4 Laboratory of Epidemiology, Republican Research Center for Radiation Medicine and Human Ecology, Gomel, Belarus; 5 Cancer Registry, State Establishment, N.N. Alexandrov National Cancer Center of Belarus, Lesnoy, Belarus; 6 School of Life Sciences, University of Sussex, Brighton, England, United Kingdom; 7 Department of General, Visceral and Thoracic Surgery, University Medical Center Hamburg-Eppendorf, Hamburg, Germany; 8 Department of Surgical Oncology and Colorectal Surgery, Brody School of Medicine, East Carolina University, Greenville, North Carolina; 9 Lung Cancer Center/Lung Cancer Institute, West China Hospital, Sichuan University, Chengdu, Sichuan, China

## Abstract

**Importance:**

To our knowledge, there are no complete population-based studies of the risks of developing second malignant tumors after papillary thyroid carcinoma (PTC) in patients following the Chernobyl nuclear accident.

**Objective:**

To study the risk of second primary cancers in patients with PTC after the Chernobyl disaster.

**Design, Setting, and Participants:**

This was a retrospective cohort study conducted in the Republic of Belarus over a 31-year time frame evaluating patients with primary PTC and second malignant tumors. Personal data from the Belarussian Cancer Registry were used in the investigation, and only second primary cancers were included in the analysis. Patients were observed from January 1, 1990, to December 31, 2021, for the establishment of second primary malignant tumors.

**Main Outcomes and Measures:**

For analysis, synchronous and metachronous tumors were grouped into 1 group (second primary cancer group). If the patient had more than 2 cancers, they were observed until development of a second tumor and, subsequently, the development of a third tumor. The starting point for calculating the number of person-years was the date of thyroid cancer diagnosis. The end point for calculating the number of person-years was the date of diagnosis of the second primary malignant tumor, the date of death, the date of the last visit of the patient, or December 31, 2021 (the end the of study period). The incidence of a second primary malignant tumor with PTC was calculated for the study groups using standardized incidence ratios.

**Results:**

Of the 30 568 patients with a primary PTC included in this study, 2820 (9.2%) developed a second malignant tumor (2204 women and 616 men); the mean (SD) age of all patients at time of the primary cancer was 53.9 (12.6) years and at time of the secondary cancer was 61.5 (11.8) years. Overall, the standardized incidence ratio was statistically significant for all types of cancer (1.25; 95% CI, 1.21-1.30), including solid malignant tumors (1.20; 95% CI, 1.15-1.25) and all leukemias (1.61; 95% CI, 2.17-2.13). Cancers of the digestive system (466 cases [21.1%]), genital organs (376 cases [17.1%]), and breasts (603 cases [27.4%]) were the most prevalent second primary tumors in women following PTC. Second primary tumors of the gastrointestinal tract (146 cases [27.7%]), genitourinary system (139 cases [22.6%]), and urinary tract (139 cases [22.6%]) were the most prevalent in men. Urinary tract cancers (307 cases [10.9%]) and gastrointestinal tumors (612 cases [21.4%]) were the most prevalent second primary tumors overall.

**Conclusions and Relevance:**

This cohort study reports the increased incidence of solid secondary tumors in men and women over a 31-year time frame after the Chernobyl disaster. Moreover, there was a statistically significant increased risk of second tumors of the breast, colon, rectum, mesothelium, eye, adnexa, meninges, and adrenal glands as well as Kaposi sarcoma. These data might have an effect on the follow-up of this cohort of patients to detect secondary malignant tumors at an early stage.

## Key Points

**Question:**

What is the risk of second primary cancers in patients with papillary thyroid carcinoma after the Chernobyl disaster?

**Findings:**

This cohort study of 30 568 patients with papillary thyroid carcinoma over a 31-year time frame found that the standardized incidence ratio was statistically significant for all cancer types, including solid malignant tumors and leukemias. Statistically significant risks of secondary tumors of the breast, colon, rectum, mesothelium, eye, adnexa, meninges, and adrenal gland as well as Kaposi sarcoma were found.

**Meaning:**

This study suggests that nuclear disasters can have substantial long-term effects requiring intense monitoring of survivors of such disasters.

## Teaser

This cohort study examines the risk of second primary cancer in patients with papillary thyroid carcinoma after the Chernobyl disaster.

## Introduction

Nearly 36 years after the Chernobyl nuclear power plant accident, there are still many unanswered concerns about the socioeconomic, environmental, and health effects of the disaster. Information from the Belarussian Cancer Registry (BCR), which recently became available for the first time, sheds light on several key Chernobyl disaster effects.

When Chernobyl nuclear power plant station No. 4 melted down on April 26, 1986, a substantial amount of radionuclides were released into the environment, causing radioactive contamination of the territories of Belarus as well as portions of Ukraine and the Russian Federation. The population’s radiation dose was mostly contributed to by 2 radionuclides: long-lived caesium-137 and short-lived iodine-131.^1^

By the method of radioecological modeling, the average doses of thyroid radiation were reconstructed for more than 9.5 million people in 19 age categories who in 1986 lived in 23 325 settlements of the Republic of Belarus. According to studies, nearly all of Belarus’s population was exposed to iodine-131 to various degrees, based on their age at the time of exposure and where they resided.^2^

In 1992, Kazakov et al^3^ provided data on the geographical distribution of thyroid cancer cases across Belarus. They showcased that after the accident, there was a statistically significant increase in the number of cases of thyroid cancer among children. According to the publication, the number of cases among children in Belarus in 1991 was 27.5 times higher than in 1986 (2 cases in 1986 vs 55 cases in 1991).

To our knowledge, the only reported health consequence associated with exposure to radioactive isotope iodine-131 after the accident was, and remains to this day, thyroid cancer in those who were a child at the time of exposure.^4^ In the adult population, the risk of thyroid cancer has been established only in Chernobyl emergency workers.^5^ However, using the BCR, Mahoney et al^6^ showed an increase in thyroid cancer rates among both sexes of all age groups. It is assumed that the high prevalence of preexisting iodine deficiency combined with exposure to iodine-131 contributed to a potential carcinogenic effect on the thyroid gland. Because there are no known thyroid radiation doses in the study population, it is impossible to determine the risk of thyroid cancer with any degree of accuracy. Over a 10-year period, there were 21.1% more thyroid cancer cases overall in the general population. In 2020, Belarus age-standardized (world) incidence was 9.0 thyroid cancer cases per 100 000 people (2.6 for men and 14.8 for women).^7,8^ In general, the accumulated risk from exposure to ionizing radiation still continues to influence the annual increase of cases of diseases to date.^4^ The incidence in the cohort of irradiated people 18 to 50 years old has stabilized but has not decreased, remaining 5 to 6 times higher than before the Chernobyl accident.^7,8^ In Belarus, after the Chernobyl disaster, papillary thyroid carcinoma (PTC) accounted for 97% of all histological cancer variants.^9^ Along with the current concern over a nuclear conflict and/or a nuclear power plant accident in a conflict zone, the current cohort study was conducted with the aim of revealing the rate of second primary malignant tumors after PTC following the Chernobyl accident to fill the knowledge gap and promote better understanding to deal with the effects of a similar event.

## Methods

### Database

Data from the BCR were used in this investigation. In the Republic of Belarus, cancer control and oncological care for the population is ensured by an extensive network of 12 oncological institutions for adult solid cancer treatment. Childhood cancer is treated at the Republican Scientific and Practical Center for Pediatric Oncology, Hematology and Immunology. Additionally, there are 2 specialized clinics for hematological pathologies. Thus, all oncological patients are treated and observed in specialized clinics, which are joined with the BCR. Every patient is observed for their entire lifespan with regular examinations by oncologists. Outcomes are tracked by polyclinics, the Census Bureau, and the Ministry of Internal Affairs. Due to this organization of the public health system, the BCR covers almost the entire population of the Republic of Belarus and tracks every patient. The BCR is the most complete information data resource on newly diagnosed and previously registered cancer in the country’s territory.

This study was approved by the ethical committee of the Republican Research Centre for Radiation Medicine and Human Ecology in Gomel, Belarus, which enables regulated access for research of anonymous data from the BCR and for which no informed consent is required. The study was performed according to the Declaration of Helsinki and followed the Strengthening the Reporting of Observational Studies in Epidemiology (STROBE) reporting guidelines.^10^

### Patients

Personal data were extracted from the BCR. Patients with primary thyroid cancer were included in the cohort. They were observed until the second cancer appeared, the time of death, the time of loss of observation, or the end of the study (December 31, 2021). Synchronous and metachronous tumors were grouped into 1 group (second primary cancer group). Second primary cancers with latency between 2 diagnoses of more than 1 year were considered metachronous, and expected numbers for them were calculated based on multiplication of person-years of observation by incidence rate. Expected numbers for synchronous cancers were calculated by multiplication of incidence rate of second tumor by number of thyroid cancer cases. If more than 2 cancers were present, patients were observed until the diagnosis of the second tumor and the subsequent following one (for example, in case of 3 cancers, person-years were calculated until the second and third, and both cancers were considered as observed numbers).

### Statistical Analysis

Standardized incidence ratios (SIRs) were calculated to assess the probability of a second primary malignant tumor. The SIRs were calculated by indirect standardization using 1-year time and 5-year age intervals (ie, 0-4, 5-9, … 75-79, ≥85 years), considering sex and place of residence. The formulas for calculating SIR are presented in the eMethods in Supplement 1.^11,12^ The calculations were made according to the method previously described by Curtis et al.^13^ Statistical analyses for demographic indicators were conducted using Prism, version 8.4.2 (GraphPad). Statistical tests were 2-sided, with *P* < .05 considered statistically significant.

## Results

### Patient Population

**Figure 1.**
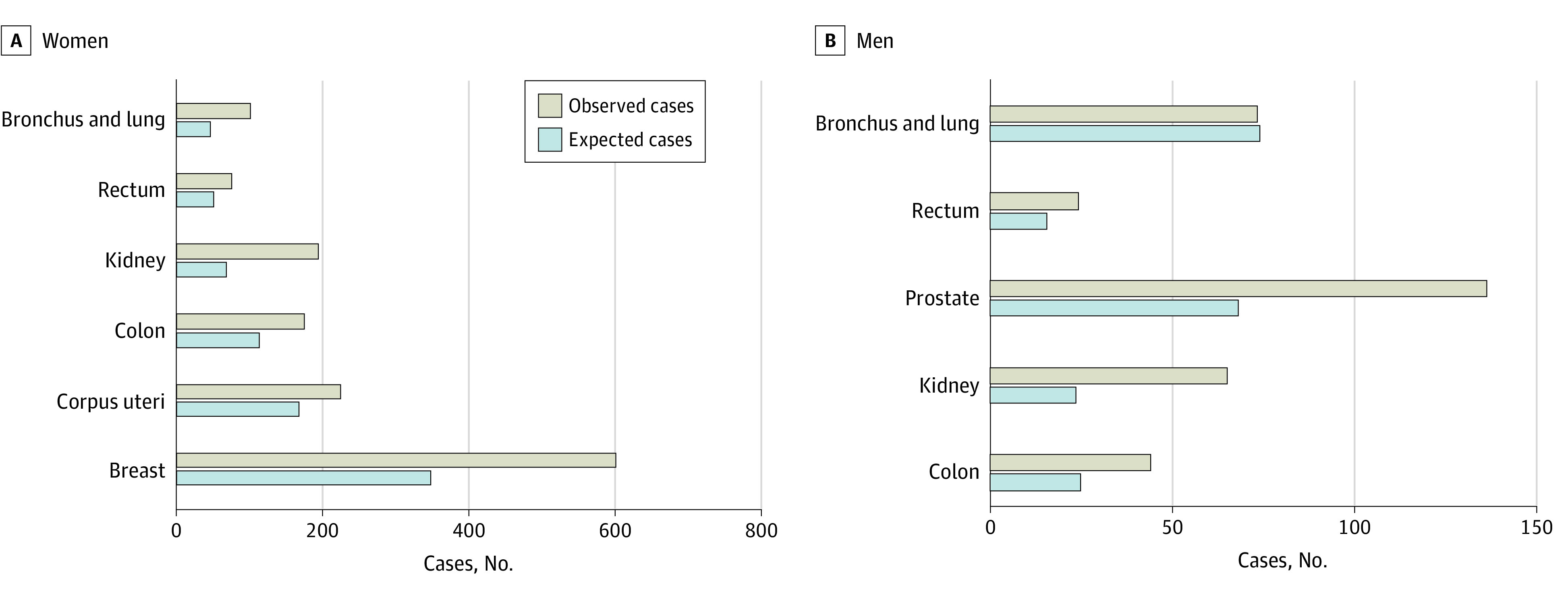
Disparity Between the Expected and Observed Number of Second Primary Malignant Tumor Localizations by Sex

**Figure 2.**
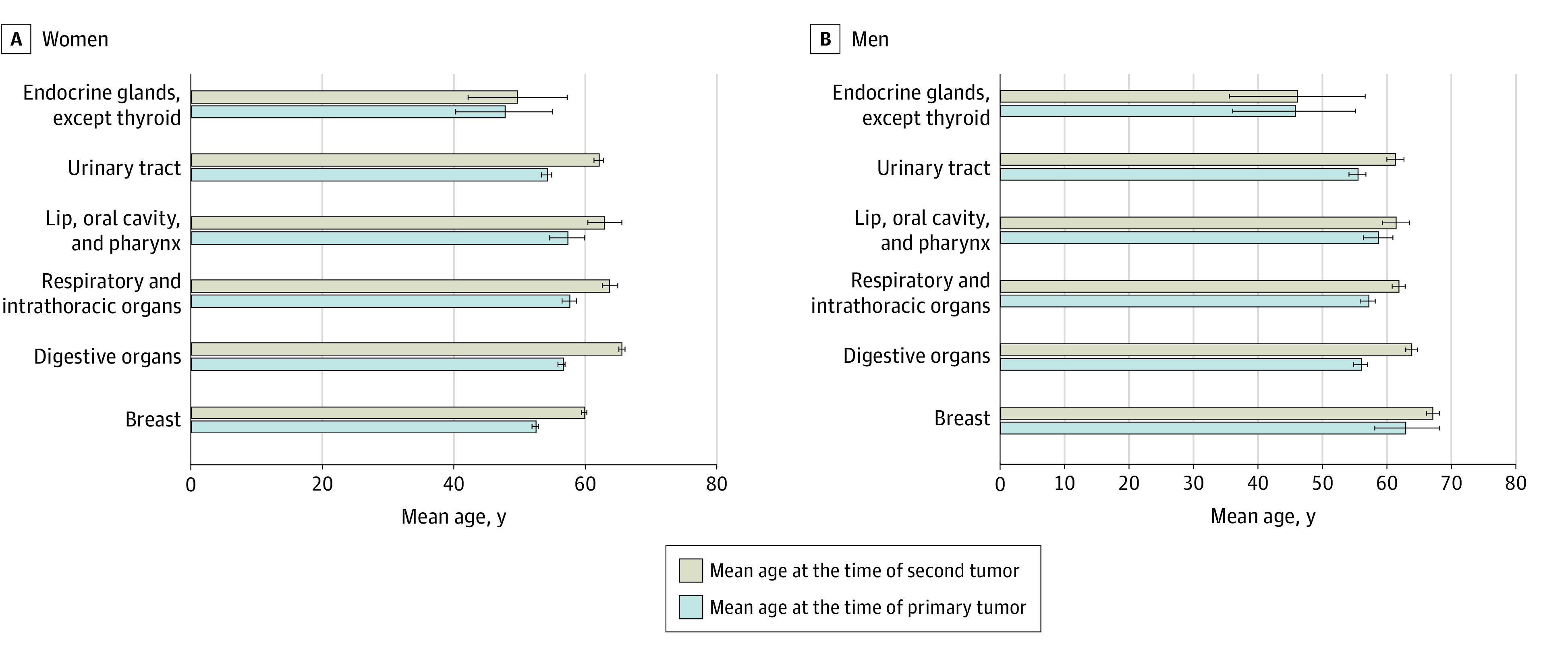
Comparison of Mean Age at the Time of Common First and Second Primary Tumors by Sex Error bars indicate 95% CIs.

In total, 30 568 patients with primary PTC were included, and 2820 second cancers were found. In women, 2204 secondary cancers occurred, and 616 occurred in men. A general overview of the distribution of second cancers according to sex is shown in Figure 1. The mean (SD) age of patients at the time of the primary cancer for both sexes was 53.9 (0.2) years and 61.5 (0.2) years at the time of secondary cancer (Figure 2).

### Risk and Type of Secondary Tumors After PTC

As summarized in eTable 1 in Supplement 1, cancers of the digestive system (466 cases [21.1%]), genital organs (376 cases [17.1%]), and breasts (603 cases [27.4%]) were the most prevalent second primary tumors in women following PTC. Second primary tumors of the gastrointestinal tract (146 cases [27.7%]), genitourinary system (139 cases [22.6%]), and urinary tract (139 cases [22.6%]) were the most prevalent in men. Urinary tract cancers (307 cases [10.9%]) and gastrointestinal tumors (612 cases [21.4%]) were the most prevalent second primary tumors overall.

#### Ear, Nose, and Throat Tumors

Women had a statistically significant higher risk than men for second primary malignant tumors of the lip, oral cavity, and pharynx (SIR, 1.72; 95% CI, 1.16-2.46). In contrast, the SIR was not statistically significant for men. However, both groups together showed a considerable increased risk of developing a second tumor at these sites (SIR, 1.45; 95% CI, 1.12-1.86).

#### Digestive System

An increased risk of developing a second primary malignant tumor of the digestive organs was found in women (SIR, 1.31; 95% CI, 1.20-1.44) and in men (SIR, 1.34; 95% CI, 1.13-1.57). There was also a statistically significant increased risk for both sexes overall (SIR, 1.32; 95% CI, 1.22-1.43).

#### Respiratory Tract

An increase in the risks of second primary malignant tumors of the respiratory organs and intrathoracic organs was detected in women (SIR, 2.23; 95% CI, 1.84-2.69). For both sexes, there was a statistically significant increased risk overall (SIR, 1.49; 95% CI, 1.29-1.71).

#### Bone, Cartilage, Mesothelium, and Soft Tissues

Only women had an increased risk of developing a second primary malignant tumor of the mesothelium and soft tissues (SIR, 2.44; 95% CI, 1.78-3.25); in contrast, SIR was not statistically significant for men. However, for both sexes overall there was an increase in the SIR (2.43; 95% CI, 1.05-4.80).

#### Genital Organs and Breasts

For second primary malignant tumors of the female and male reproductive systems and breasts, there was an increased risk of female breast cancer (SIR, 1.72; 95% CI, 1.58-1.86). Women also had an increased risk of cancers of the reproductive system (SIR, 1.12; 95% CI, 1.01-1.24), as did men (SIR, 1.95; 95% CI, 1.64-2.31).

#### Urinary Tract

The SIRs for urinary tract tumors showed increased risks in women (2.47; 95% CI, 2.16-2.82) and men (2.04; 95% CI, 1.64-2.52). The only malignant tumor among all of the individuals in this group with statistically significant increased risk was kidney cancer (SIR, 2.79; 95% CI, 2.46-3.15).

#### Lymphoid and Hematopoietic Systems

Second primary malignant tumors of lymphoid, hematopoietic, and associated tissue had the highest risk for second primary malignant tumors. Overall, this risk was elevated (SIR, 2.24; 95% CI, 1.97-2.54). The SIR values for individual types of tumors are summarized in eTable 2 in Supplement 1.

#### Skin Tumors and Kaposi Sarcoma

Overall, there was an increased risk of melanoma (SIR, 2.21; 95% CI, 1.79-2.71). In both men (SIR, 3.16; 95% CI, 1.87-4.99) and women (SIR, 2.07; 95% CI, 1.63-2.59) this risk remained elevated. Women had an increased risk for Kaposi sarcoma (SIR, 6.26; 95% CI, 1.29-18.28) and minor neoplasm of other connective and soft tissue (SIR, 2.90; 95% CI, 1.84-4.34), while in men this risk was not statistically significantly elevated.

#### Eye and Central Nervous System

The overall SIR for malignant neoplasm of the eye and adnexa was elevated (SIR, 2.26; 95% CI, 1.24-3.79). In men the risk was not elevated; however, there was increased risk in women for individual localized cancers in these sites. Only women were at risk for meningeal neoplasms (SIR, 3.65; 95% CI, 1.19-8.52). Men (SIR, 2.27; 95% CI, 1.13-4.06) and both sexes combined (SIR, 1.43; 95% CI, 1.01-1.98) had a higher chance of developing brain cancer. Overall, there was a statistically significant difference (SIR, 4.00; 95% CI, 1.09-10.24) for developing a spinal cord and cranial nerve malignant neoplasm.

#### Endocrine System

Only women had an increased risk for malignant neoplasm of other endocrine glands and related structures (SIR, 17.77; 95% CI, 3.66-51.92). Overall, there was a statistically significant increased risk for adrenal tumors (SIR, 3.17; 95% CI, 1.03-7.40) and malignant neoplasm of other endocrine glands and related structures (SIR, 14.69; 95% CI, 3.03-42.92).

### Age at First Tumor (PTC)

As summarized in eTable 3 in Supplement 1, the mean (SD) age of the first tumor for all malignant neoplasms overall was 53.9 (0.2) years. Women presented slightly earlier in life than men (mean [SD] age, 53.3 [0.3] years vs 56.2 [0.5] years). Overall, the mean (SD) age at diagnosis for solid tumors was 54.0 (0.3) years (women, 53.3 [0.3] years; men, 56.4 [0.5] years) and for all leukemias was 53.4 (1.2) years (women, 53.5 [1.3] years; men, 53.1 [3.2] years).

### Age at Second Tumor

Overall, the mean (SD) age at second tumor diagnosis was 61.5 (0.2) years (women, 61.2 [0.3] years; men, 62.6 [0.5] years). For all solid tumors, the mean (SD) age at diagnosis was 61.6 (0.2) years (women, 61.2 [0.3] years; men, 62.8 [0.5] years). Mean (SD) age was similar at diagnosis for all leukemias (overall, 60.6 [1.2] years; women, 61.4 [1.3] years; men, 58.3 [2.9] years). For individual sites for the second tumor, the most advanced mean (SD) age for both sexes were for tumors of ill-defined sites, secondary and unspecified sites (67.7 [1.6] years), and digestive organs (65.0 [0.5] years).

### Latency Between PTC and Second Tumor

For individual sites, the latency period in mean (SD) years was longest for both sexes for tumors of the digestive organs (8.9 [0.3] years) and tumors of ill-defined, secondary, and unspecified sites (8.0 [1.3] years); tumors of the endocrine glands (1.7 [0.9] years) and tumors of respiratory and intrathoracic organs (5.6 [0.4] years) had the shortest latency period (Figure 2).

## Discussion

This study presents a comprehensive report of the risk of developing a second primary malignant tumor among adult patients with PTC in the overall time frame of 31 years after the Chernobyl disaster. After treatment of thyroid cancer in the years following the Chernobyl accident, second primary malignant tumors were found to have a statistically significant increased risk of development. Previous publications have reported an increased risk of both solid tumors and leukemia.^14,15,16,17,18,19^ Prior to the current study, only 2 reports were found on the development of a second primary malignant tumor after thyroid cancer.^20,21^ In a cohort of 6559 patients with post-Chernobyl PTC, an increased risk was demonstrated in patients with solid secondary malignant tumors in general and in the colon, female breast, and urinary system.^21^ Additionally, an increased risk was found in patients with leukemia, which most likely reflects radiation exposure to the bone marrow. All patients were children at the time of the Chernobyl accident. Accordingly, PTC was detected at a median age of 29 (range, 23-36) years.

This analysis has demonstrated an increased risk of second primary tumors, with some differences between sexes, as the risk for second tumors of all sites, including solid cancers and all leukemias, was statistically significant for both sexes. In women, there was a statistically significant elevated risk of second primary malignant tumors of the breast after PTC (603 cases; 27.4% of all second tumors), as well as a statistically significant elevated risk of second primary malignant tumors of the parotid gland, oropharynx, larynx, trachea, and bronchi. These results correspond to previous studies for US and Asian populations.^22,23^ It is interesting that women were shown to be at higher risk for these tumor types, as smoking was thought to raise the risk of some cancers in men, such as tumors of the oral cavity, oropharynx, larynx, trachea, bronchi, and lungs.

The present study also showed an increased risk for second primary malignant tumors of the digestive organs in women, including colon and rectal cancer. Additionally, men showed a higher risk of gastrointestinal tumors, including colon cancer. There have been reports of an increased risk of small and large bowel cancers; however, the individuals in these investigations had various postoperative treatments (radiation therapy, radioisotopes, as well as other forms of treatment). Moreover, different histological types of thyroid cancer were examined in addition to PTC.^14,19,23^ In a cohort of children exposed to iodine-131 at the time of the Chernobyl accident, only 1 report showed an increased risk of second colon cancer.^21^ The results of the present study on secondary tumors after PTC were not shown earlier for such a large population.

The current analysis also found that the group of soft tissue tumors, the risks for mesothelioma, and Kaposi sarcoma were increased, which to our knowledge has not been previously described. Also, the risk of secondary tumors of the eye and adnexa, meninges, and the adrenal gland have not been previously described in women. For malignant tumors of the lymphoid, hematopoietic, and related tissue, increased risks are shown in both sexes. Furthermore, and to our knowledge, the increased risk for Hodgkin lymphoma has not been previously described.

It is known that the radioiodine treatment of PTC increases the risk of second primary malignant tumors, especially bone cancer, kidney cancer, hematological cancers, and prostate cancer, as shown in numerous studies.^14,16,22,24,25^ Tumors of the salivary glands had the highest risk followed by bone and joint tumors and chronic myeloid leukemia. Interestingly, the incidence of colorectal cancer was found to be lower in survivors of thyroid cancer compared with the general population.^19^

### Limitations

The main limitation of this study is that the cumulative radiation dose that the individuals absorbed is not considered for the lack of such data. To obtain these doses by radioecological modeling methods, additional data sources would be needed (thyroid gland and thyroid gland mass; measurements of the dose rate of iodine-131 in soil, grass samples, and food; and other variables and data points). Considering all of these difficulties, we could not analyze the contribution of radiation doses to the development of second primary malignant tumors in individual patients.

Furthermore, to minimize immortal bias, we calculated expected numbers of synchronous cancers by multiplication of incidence rates. We supposed that synchronous cancer is diagnosed more often due to more accurate patient examination because of first tumor. Thus, we did not know which tumor was the first. Therefore, the number of person-years could not be accurately determined and, as such, the probability of occurrence of synchronous tumors was defined as the product of the multiplication of the first and second tumors.

## Conclusions

The results of this cohort study indicate a statistically significant elevated incidence of solid secondary tumors over a 31-year time frame for the first time, to our knowledge. Statistically significant risks of secondary tumors of the breast, colon, rectum, mesothelium, eye, adnexa, meninges, and adrenal gland as well as Kaposi sarcoma were also described, to our knowledge, for the first time in a population-based investigation including patients with PTC following the Chernobyl accident. These data might have an effect on the follow-up of this cohort of patients to detect secondary malignant tumors at an early stage. This study shows that nuclear disasters can have substantial long-term effects requiring intense monitoring of victims of such disasters.

## Notes

Supplement 1.
eMethods.
**eTable 1.** SIR for second primary malignant tumors after papillary thyroid cancer **eTable 2.** SIR for second primary malignant tumors for localizations after papillary thyroid cancer **eTable 3.** Age and latency period between the first and second tumors (Mean±SE)

Supplement 2.
Data Sharing Statement
