# Supplementary material for: Second Primary Cancer Among Patients With Papillary Thyroid Carcinoma Following the Chernobyl Disaster
Source: JAMA Netw Open. 2023 Aug 17;6(8):e2329559. doi: 10.1001/jamanetworkopen.2023.29559 (PMC10436126; doi:10.1001/jamanetworkopen.2023.29559)
Supplement: Supplement 1. — eMethods. eTable 1. SIR for second primary malignant tumors after papillary thyroid cancer eTable 2. SIR for second primary malignant tumors for localizations after papillary thyroid cancer eTable 3. Age and latency period between the first and second tumors (Mean±SE) [file jamanetwopen-e2329559-s001.pdf]

Supplemental Online Content

Taha A, Taha-Mehlitz S, Nadyrov EA, et al. Second primary cancer among patients with papillary thyroid carcinoma following the Chernobyl disaster. *JAMA Netw Open*. 2023;6(8):e2329559. doi:10.1001/jamanetworkopen.2023.29559

eMethods.

eTable 1. SIR for second primary malignant tumors after papillary thyroid cancer

eTable 2. SIR for second primary malignant tumors for localizations after papillary thyroid cancer

eTable 3. Age and latency period between the first and second tumors (Mean±SE)

This supplemental material has been provided by the authors to give readers additional information about their work.

© 2023 Taha A et al. *JAMA Network Open.*

eMethods.

The formulas for calculating Standardized incidence ratios (SIR)

The SIR of synchronous and metachronous second primary malignant tumor is the ratio of the observed number of events (a certain combination of tumors) to the expected number of second cancer, calculated for a certain number of person-years, based on population levels of cancer incidence rates (formula (1)):

$$SIR = \frac{Obs.m + Obs.s}{Exp.m + Exp.s} \qquad (1)$$

SIR – Standardized incidence ratio;

Obs. – Observed number of certain cancer combination (m – metachronous and s – synchronous);

Exp. – Expected number of certain cancer combination (m – metachronous and s – synchronous);

Expected numbers of certain cancer combination of synchronous malignancies were calculated by formula (2):

$$Exp.s. = \sum_i \sum_j \frac{n_{ij}^{(thyroid)} \times n_{ij}^{(\sec ond)}}{N_{ij}} \qquad (2)$$

$n^{(thyroid)}_{ij}$– the number of cases of thyroid cancer in the population of Belarus, in the j-th age group and the i-th calendar time interval;

$n^{(second)}_{ij}/N_{ij}$ – the age-specific rate of cancer incidence with localization corresponding to the second studied tumor in the population of the Republic of Belarus, in the j-th age group and the i-th calendar time interval; The expected numbers of metachronous tumors was calculated by the formula (3):

$$Exp.m. = \sum_i \sum_j \frac{pyrs_{ij}^{(thyroid)} \times n_{ij}^{(\sec ond)}}{N_{ij}} \qquad (3)$$

$pyrs^{(thyroid)}_{ij}$– the number of person-years in a cohort of patients with thyroid cancer, in the j-th age group and the i-th calendar time interval;

$n^{(second)}_{ij}/N_{ij}$ – age-specific incidence rate of localization corresponding to the second studied tumor in the population of the Republic of Belarus, in the j-th age group and the i-th calendar time interval;

The calculation of SIR was based on comparing the number of detected cases of thyroid cancer with a certain combination of localizations with the calculated expected number based on incidence of malignant tumors after the Chernobyl disaster. We assumed that the appearance of multiple tumors in the same person are independent events, and their incidence corresponds to the product of the incidence of individual tumors in the corresponding group of the population of Belarus. Assuming Obs. is Poisson distributed with mean μ = E(Obs.), confidence limits for μ are obtained using the relationship between the Poisson distribution and the chi-square distribution. To assess the statistical significance of the SIR, exact 95% confidence intervals (CI^- and CI^+) were calculated (4):

$$CI^- = \frac{\chi^2_{(2Obs.,\frac{\alpha}{2})}}{2Exp.} \qquad CI^+ = \frac{\chi^2_{(2(Obs.+1),1-\frac{\alpha}{2})}}{2Exp.} \qquad (4)$$

$\chi_{v,\alpha}{}^2$ is the 100α percentile of the chi-square distribution with *v* degrees of freedom.

When the lower bound of the 95% CI- is higher than 1.0, the risk is considered significantly elevated at the level P = .05”. Statistical analyses for demographic indicators were conducted using GraphPad prism 8.4.2. Statistical tests were two-sided, with P < .05 considered statistically significant.

© 2023 Taha A et al. *JAMA Network Open.*

eTable 1. SIR for second primary malignant tumors after papillary thyroid cancer

All sites	Women					Men					Both sexes				
	EXP	OBS	SIR	95%CI-	95%CI+	EXP	OBS	SIR	95%CI-	95%CI+	EXP	OBS	SIR	95%CI-	95%CI+
**All malignant neoplasms**	1810.29	2204	1.22*	1.17	1.27	441.07	616	1.40*	1.29	1.51	2251.36	2820	1.25*	1.21	1.30
**Solid tumors**	1703.09	1994	1.17*	1.12	1.22	412.94	545	1.32*	1.21	1.44	2116.03	2539	1.20*	1.15	1.25
**Lip, oral cavity and pharynx**	17.40	30	1.72*	1.16	2.46	25.22	32	1.27	0.87	1.79	42.63	62	1.45*	1.12	1.86
**Digestive organs**	354.72	466	1.31*	1.20	1.44	109.13	146	1.34*	1.13	1.57	463.85	612	1.32*	1.22	1.43
**Respiratory and intrathoracic organs**	50.57	113	2.23*	1.84	2.69	87.04	92	1.06*	0.85	1.30	137.61	205	1.49*	1.29	1.71
**Bone and articular cartilage**	2.54	6	2.36	0.87	5.14	0.75	2	2.68	0.32	9.69	3.29	8	2.43*	1.05	4.80
**Melanoma**	37.21	77	2.07*	1.63	2.59	5.70	18	3.16*	1.87	4.99	42.92	95	2.21*	1.79	2.71
**Mesothelial and soft tissue**	18.88	46	2.44*	1.78	3.25	3.52	8	2.27	0.98	4.48	22.40	54	2.41*	1.81	3.14
**Breast**	350.95	603	1.72*	1.58	1.86	0.57	2	3.51	0.42	12.67	351.52	605	1.72*	1.59	1.86
**Female genital organs**	336.51	376	1.12*	1.01	1.24	-	-	-	-	-	336.51	376	1.12*	1.01	1.24
**Male genital organs**	0.00	-	-	-	-	71.15	139	1.95*	1.64	2.31	71.15	139	1.95*	1.64	2.31
**Urinary tract**	88.95	220	2.47*	2.16	2.82	42.60	87	2.04*	1.64	2.52	131.54	307	2.33*	2.08	2.61
**Eye, brain and other parts of CNS**	27.82	45	1.62*	1.18	2.16	6.04	14	2.32*	1.27	3.89	33.87	59	1.74*	1.33	2.25
**Endocrine glands, except thyroid**	1.38	6	4.35*	1.60	9.48	0.40	2	4.97	0.60	17.96	1.78	8	4.49*	1.94	8.85
**Ill-defined, secondary and unspecified sites**	20.15	32	1.59*	1.09	2.24	7.67	8	1.04	0.45	2.05	27.82	40	1.44*	1.03	1.96
**Lymphoid, haematopoietic and related tissue**	87.05	178	2.04*	1.76	2.37	20.46	63	3.08*	2.37	3.94	107.51	241	2.24*	1.97	2.54
**All leukemias**	37.14	91	2.45*	1.97	3.01	9.60	31	3.23*	2.19	4.59	46.74	122	2.61*	2.17	3.12

EXP – expected cases; OBS – observed cases; SIR – standardized incident ratio; CI – confidence interval (95%); * p value <0.05.

© 2023 Taha A et al. *JAMA Network Open.*

eTable 2. SIR for second primary malignant tumors for localizations after papillary thyroid cancer

All sites	Women					Men					Both sexes				
	EXP	OBS	SIR	95%CI-	95%CI+	EXP	OBS	SIR	95%CI-	95%CI+	EXP	OBS	SIR	95%CI-	95%CI+
**Parotid gland**	1.92	5	2.60	0.84	6.07	0.62	2	3.22	0.39	11.64	2.54	7	2.75*	1.11	5.67
**Oropharynx**	1.10	3	2.74	0.56	8.00	3.64	8	2.20	0.95	4.33	4.74	11	2.32*	1.16	4.16
**Colon**	114.07	176	1.54*	1.32	1.79	24.56	44	1.79*	1.30	2.40	138.63	220	1.59*	1.38	1.81
**Rectum**	51.83	75	1.45*	1.14	1.81	15.46	24	1.55	0.99	2.31	67.29	99	1.47*	1.20	1.79
**Larynx**	1.77	7	3.94*	1.59	8.13	11.97	17	1.42	0.83	2.27	13.74	24	1.75*	1.12	2.60
**Trachea**	0.26	2	7.61	0.92	27.50	0.25	1	3.98	0.10	22.18	0.51	3	5.84*	1.20	17.06
**Bronchus and lung**	46.69	101	2.16*	1.76	2.63	73.95	73	0.99	0.77	1.24	120.64	174	1.44*	1.24	1.67
**Bone and articular cartilage of limbs**	1.15	3	2.62	0.54	7.65	0.34	2	5.86	0.71	21.18	1.49	5	3.36*	1.09	7.85
**Melanoma**	37.21	77	2.07*	1.63	2.59	5.70	18	3.16*	1.87	4.99	42.92	95	2.21*	1.79	2.71
**Mesothelioma**	2.01	8	3.98*	1.72	7.84	0.59	1	1.70	0.04	9.45	2.60	9	3.46*	1.58	6.57
**Kaposi sarcoma**	0.48	3	6.26*	1.29	18.28	0.28	0	0.00	0.00	13.30	0.76	3	3.96	0.82	11.58
**Other connective and soft tissue**	7.94	23	2.90*	1.84	4.34	1.77	6	3.40*	1.25	7.40	9.71	29	2.99*	2.00	4.29
**Breast**	350.95	603	1.72*	1.58	1.86	0.57	2	3.51	0.42	12.67	351.52	605	1.72*	1.59	1.86
**Corpus uteri**	169.40	225	1.33*	1.16	1.51	0.00	0	-	0.00	-	169.40	225	1.33*	1.16	1.51
**Prostate**	0.00	0	-	0.00	-	68.19	136	1.99*	1.67	2.36	68.19	136	1.99*	1.67	2.36
**Kidney**	69.33	194	2.80*	2.42	3.22	23.54	65	2.76*	2.13	3.52	92.87	259	2.79*	2.46	3.15
**Eye and adnexa**	5.31	12	2.26*	1.17	3.95	0.89	2	2.26	0.27	8.16	6.20	14	2.26*	1.24	3.79
**Meninges**	1.37	5	3.65*	1.19	8.52	0.15	0	0.00	0.00	25.26	1.52	5	3.30*	1.07	7.70
**Brain**	20.31	25	1.23	0.80	1.82	4.84	11	2.27*	1.13	4.06	25.15	36	1.43*	1.01	1.98
**Spinal cord, cranial nerves**	0.83	3	3.61	0.74	10.54	0.17	1	5.95	0.15	33.17	1.00	4	4.00*	1.09	10.24
**Adrenal gland**	1.21	3	2.48	0.51	7.25	0.37	2	5.45	0.66	19.69	1.58	5	3.17*	1.03	7.40
**Other endocrine glands**	0.17	3	17.77*	3.66	51.92	0.04	0	0.00	0.00	104.23	0.20	3	14.69*	3.03	42.92

© 2023 Taha A et al. *JAMA Network Open.*

**Hodgkin lymphoma**	7.53	16	2.13*	1.22	3.45	1.87	8	4.28*	1.85	8.43	9.40	24	2.55*	1.64	3.80
**Follicular lymphoma**	2.20	7	3.18*	1.28	6.56	0.43	1	2.31	0.06	12.86	2.63	8	3.04*	1.31	5.99
**Non-follicular lymphoma**	14.01	25	1.78*	1.15	2.63	3.11	10	3.22*	1.54	5.92	17.12	35	2.04*	1.42	2.84
**Other and unspecified types of non-Hodgkin lymphoma**	10.40	11	1.06	0.53	1.89	2.42	8	3.30*	1.43	6.50	12.82	19	1.48	0.89	2.31
**Multiple myeloma and plasma cell neoplasms**	13.88	25	1.80*	1.17	2.66	2.47	4	1.62	0.44	4.15	16.35	29	1.77*	1.19	2.55
**Lymphoid leukaemia**	19.09	47	2.46*	1.81	3.27	5.58	20	3.59*	2.19	5.54	24.67	67	2.72*	2.11	3.45
**Myeloid leukaemia**	12.75	32	2.51*	1.72	3.54	2.77	9	3.25*	1.48	6.16	15.52	41	2.64*	1.90	3.58
**Other neoplasms of lymphoid, haemato-poietic and related tis-sue**	0.19	2	10.54*	1.28	38.06	0.05	0	0.00	0.00	81.22	0.24	2	8.50*	1.03	30.71

© 2023 Taha A et al. *JAMA Network Open.*

eTable 3. Age and latency period between the first and second tumors (Mean±SE)

All sites	Women			Men			Both sexes		
	Age of the first tumor	Age of the second tumor	Latency Period in years	Age of the first tumor	Age of the second tumor	Latency Period in years	Age of the first tumor	Age of the second tumor	Latency Period in years
**All malignant neoplasms**	53.3±0.27	61.2±0.25	7.9±0.15	56.2±0.52	62.6±0.48	6.4±0.27	53.9±0.24	61.5±0.22	7.6±0.13
**Solid tumors**	53.3±0.28	61.2±0.26	8.0±0.15	56.4±0.52	62.8±0.47	6.5±0.29	54.0±0.24	61.6±0.23	7.7±0.13
**Lip, oral cavity and pharynx**	57.1±2.69	62.8±2.59	5.6±1.13	58.5±2.29	61.3±2.08	2.9±0.94	57.9±1.75	62±1.64	4.2±0.74
**Digestive organs**	56.3±0.55	65.4±0.51	9.2±0.32	55.8±1.08	63.7±0.92	8±0.62	56.2±0.49	65±0.45	8.9±0.29
**Respiratory and intrathoracic organs**	57.4±1.12	63.6±1.12	6.2±0.56	56.9±1.16	61.7±1.0	4.9±0.65	57.2±0.81	62.8±0.77	5.6±0.43
**Bone and articular cartilage**	52.8±4.32	58.5±4	5.9±2.18	46.5±18.5	53.5±25.5	6.8±6.77	51.3±4.82	57.3±5.7	6.1±2.05
**Melanoma**	52.0±1.76	59±1.65	7.1±0.79	55.9±1.91	61.2±1.98	5.5±1.81	52.7±1.48	59.4±1.39	6.8±0.72
**Mesothelial and soft tissue**	51.0±2.21	58.1±2.16	7.1±0.84	45.0±5.87	51.1±3.09	6.2±3.2	50.1±2.07	57.1±1.92	7.0±0.85
**Breast**	52.2±0.5	59.7±0.45	7.6±0.29	63.0±5.0	67.0±1.0	4.4±4.42	52.3±0.5	59.7±0.45	7.5±0.29
**Female genital organs**	49.8±0.6	58±0.55	8.3±0.36	-	-	-	49.8±0.6	58±0.55	8.3±0.36
**Male genital organs**	-	-	-	59.0±0.96	66.4±0.79	7.5±0.54	59±0.96	66.4±0.79	7.5±0.54
**Urinary tract**	54.0±0.75	61.9±0.74	8.0±0.46	55.3±1.32	61.2±1.32	6.1±0.73	54.4±0.66	61.7±0.65	7.5±0.39
**Eye, brain and other parts of CNS**	50.5±1.63	58.4±1.55	7.8±0.95	45±2.54	50.4±3.26	5.6±1.34	49.2±1.41	56.5±1.47	7.3±0.79
**Endocrine glands, except thyroid**	47.5±7.33	49.5±7.53	2.1±1.11	45.5±9.5	45.5±10.5	0.6±0.4	47±5.67	48.5±5.89	1.7±0.85
**Ill-defined, secondary and unspecified sites**	60.4±1.9	68.6±1.62	8.2±1.55	57.1±5.1	64.1±4.99	7.1±2.28	59.7±1.81	67.7±1.62	8.0±1.31
**Lymphoid, haemato-poietic and related tis-sue**	53.4±1.0	60.7±1.03	7.4±0.52	53.5±2.2	58.7±2.07	5.2±0.68	53.4±0.93	60.2±0.94	6.8±0.43
**All leukemias**	53.5±1.27	61.4±1.32	8.0±0.73	53.1±3.16	58.3±2.9	5.3±0.85	53.4±1.24	60.6±1.24	7.3±0.6

© 2023 Taha A et al. *JAMA Network Open.*
