# Supplementary material for: Second Primary Cancer Among Patients With Papillary Thyroid Carcinoma Following the Chernobyl Disaster
Source: JAMA Netw Open. 2023 Aug 17;6(8):e2329559. doi: 10.1001/jamanetworkopen.2023.29559 (PMC10436126; doi:10.1001/jamanetworkopen.2023.29559)
Supplement: Supplement 2. — Data Sharing Statement [file jamanetwopen-e2329559-s002.pdf]

Data Sharing Statement

Taha. Second Primary Cancer Among Patients With Papillary Thyroid Carcinoma Following the Chernobyl Disaster. *JAMA Netw Open*. Published August 17, 2023.
doi:10.1001/jamanetworkopen.2023.29559

Data
Data available: No

Additional Information
Explanation for why data not available: Data will be made available upon reasonable request.
